# Supplementary material for: One-Cell Doubling Evaluation by Living Arrays of Yeast, ODELAY!
Source: G3 (Bethesda). 2016 Nov 16;7(1):279–88. doi: 10.1534/g3.116.037044 (PMC5217116; doi:10.1534/g3.116.037044)
Supplement: Supplementary file 1 [file 279FigureS1.pdf]

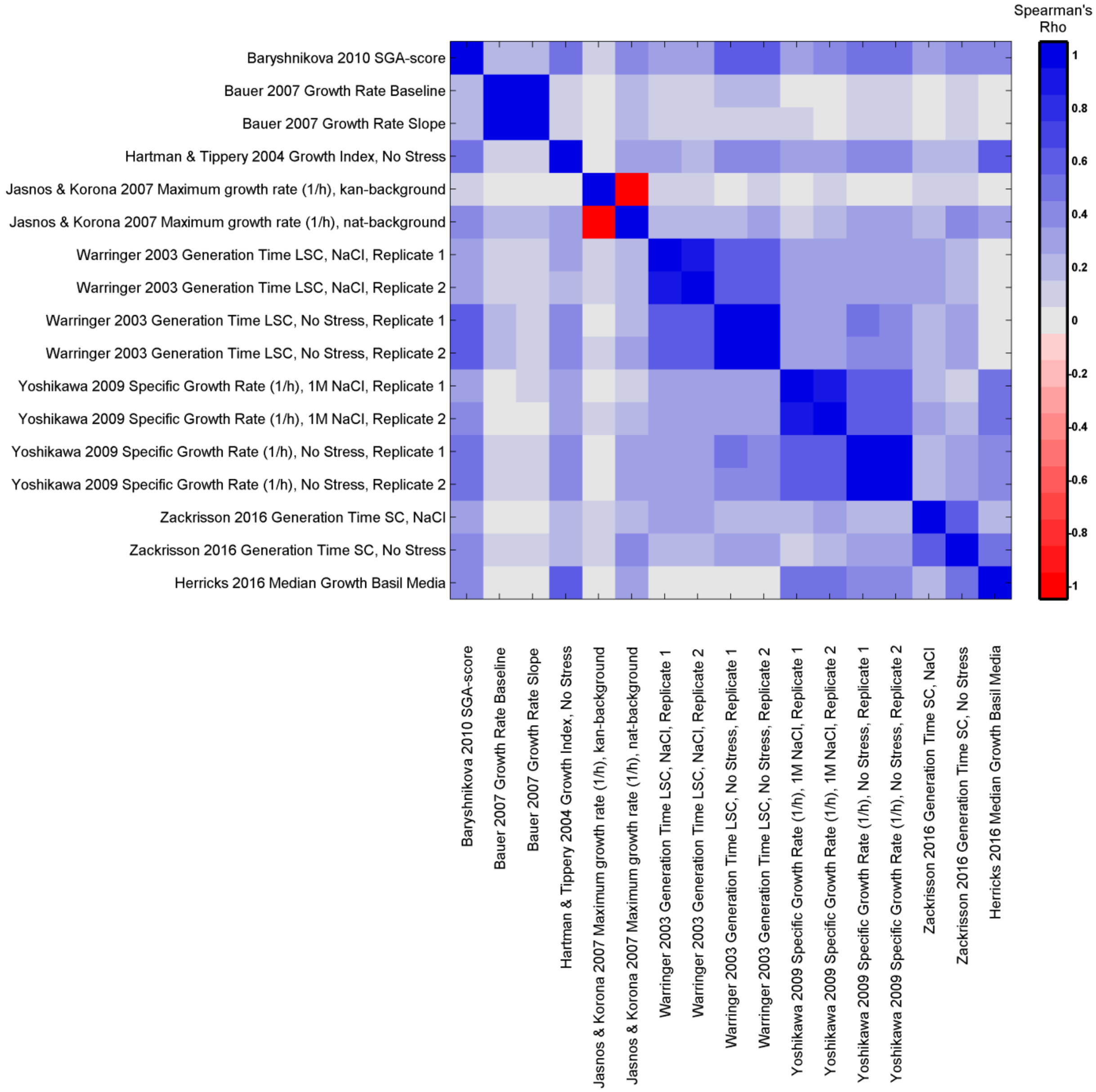

Figure S1: Spearman's Rho rank correlation coefficient with other previously published studies. This figure was generated with data originally published elsewhere and generously shared for by M. Zackrisson and A. Blomberg (Zackrisson et al., 2016). The ODELAY data set is relatively small but shows modest correlations with other datasets. Overall correlation
