# Supplementary material for: One-Cell Doubling Evaluation by Living Arrays of Yeast, ODELAY!
Source: G3 (Bethesda). 2016 Nov 16;7(1):279–88. doi: 10.1534/g3.116.037044 (PMC5217116; doi:10.1534/g3.116.037044)
Supplement: Supplementary file 2 [file 279FileS1.zip › ODELAY Hardware Design and Protocol/ODELAY Experiment Protocol v1.docx]

**ODELAY Experiment Outline:**

1. Prepping Agarose Gels
2. Pouring ODELAY Agarose Gels
3. Preculture plate over night
4. First dilution Hamilton Robot
5. Clean and prepping ODELAY Stage inserts
6. Second dilution after ~4-5hrs
7. Spotting on Agar
8. Setting up and running ODELAY
9. **Prepping Agaros Gels**

Reagents

High purity agarose

Ultra pure sterile water

9x 50mL Falcon tubes

1x 500 mL screw cap media bottle

1x scale 0.00g resolution

1x 25mL pippet

Notes: Please perform all measurements by mass in these steps as evaporation and agarose temperature can cause changes in volume and ultimately concentration of agarose.

In 500mL bottle

1. Weigh 2g of high purity agarose
2. Weigh bottle + 2g agarose
3. Calculate target weight of bottle +2g agarose weight +150g
4. **Weigh out 150g of ultra pure water within 0.1 g or better**
5. Note weight of bottle + agarose + water
6. Microwave bottle with cap placed on top (very loose not tight) to reduce water evaporation
7. After boiling water and ensuring all agarose is melted
8. Weigh flask and add ultra pure water to within 0.1g of noted weight
9. Swirl to mix new water and ensure agar is homogenious
10. Using scale Aliquot ~15.2 grams into 9x 50mL Falcon tubes.
11. **Pouring ODELAY Agarose Gels:**

**CSM Dropout Gels**

**Reagents:**

70% EtOH spray bottle.

10X CSM media

100X Adenine ADE 1 mg/ml

100X Lysine LYS 10 mg/ml

100X Tryptophan TRP 10 mg/ml

100X Leucine LEU 10 mg/ml

100X Histadine HIS 5 mg/ml

20wt% glucose 10x carbons source reagent

20wt% rafinose reagent

20wt% galactose reagent

**Solid Media Preparation:**

1. Clean 4x 2x3 inch 1mm thick glass slides with ethanol and dried using airgun
2. Clean acrylic molds with ethanol, dry and assemble as shown
3. Assembled spacers and glass slides and placed on protein gel cast mount
4. Add 2mL of 10X media (YPD, YPD, CSM)
5. Add additional reagents or water to bring total volume* to ~18mL
6. Weigh 1% agarose aliquot in 50mL tube before placing in boiling water
7. Boil 16 minutes vortex and replace in boiling water for 2 more minutes
8. Weigh tube and replace add ultra pure sterile water to replace mass ~70μL
9. Add 2mL of 10x carbon source reagent and vortex
10. Take 70 μL and fill gaps in sides of glass assembly
11. Fill ~6.5-7mL of agarose into molds
12. Wat 40-50 minutes for agar to solidify
13. Store plates 4C humidified box overnight (tip box with a bit of water in the bottom)

**YEP Gels**

**Reagents:**

70% EtOH spray bottle.

10X YEP media

10X Carbon source reagent

20wt% glucose 10x carbons source reagent

20wt% rafinose reagent

20wt% galactose reagent

Sterile Pure Water

**Solid Media Preparation:**

1. Clean 4x 2x3 inch 1mm thick glass slides with ethanol and dry using airgun
2. Clean acrylic molds with ethanol, dry and assemble as shown
3. Assembled spacers and glass slides and placed on protein gel cast mount (see Images)
4. Add 2mL of 10X media (YPD, YPD, CSM)
5. Add additional reagents or water to bring total volume* to ~18mL
6. Weigh 1% agarose aliquot in 50mL tube before placing in boiling water
7. Boil 16 minutes vortex to homogenize mixture and replace in boiling water for 2 more minutes
8. Weigh tube and replace add ultra pure sterile water to replace mass ~70μL
9. Add 2mL of 10x carbon source reagent and vortex
10. Take 70 μL and fill gaps in sides of glass assembly
11. Fill ~6.5-7mL of agarose into molds
12. Wat 40-50 minutes for agar to solidify
13. Store plates 4C humidified box overnight (tip box with a bit of water in the bottom)

**Separation of Glass Slides:**

**Notes on**

| **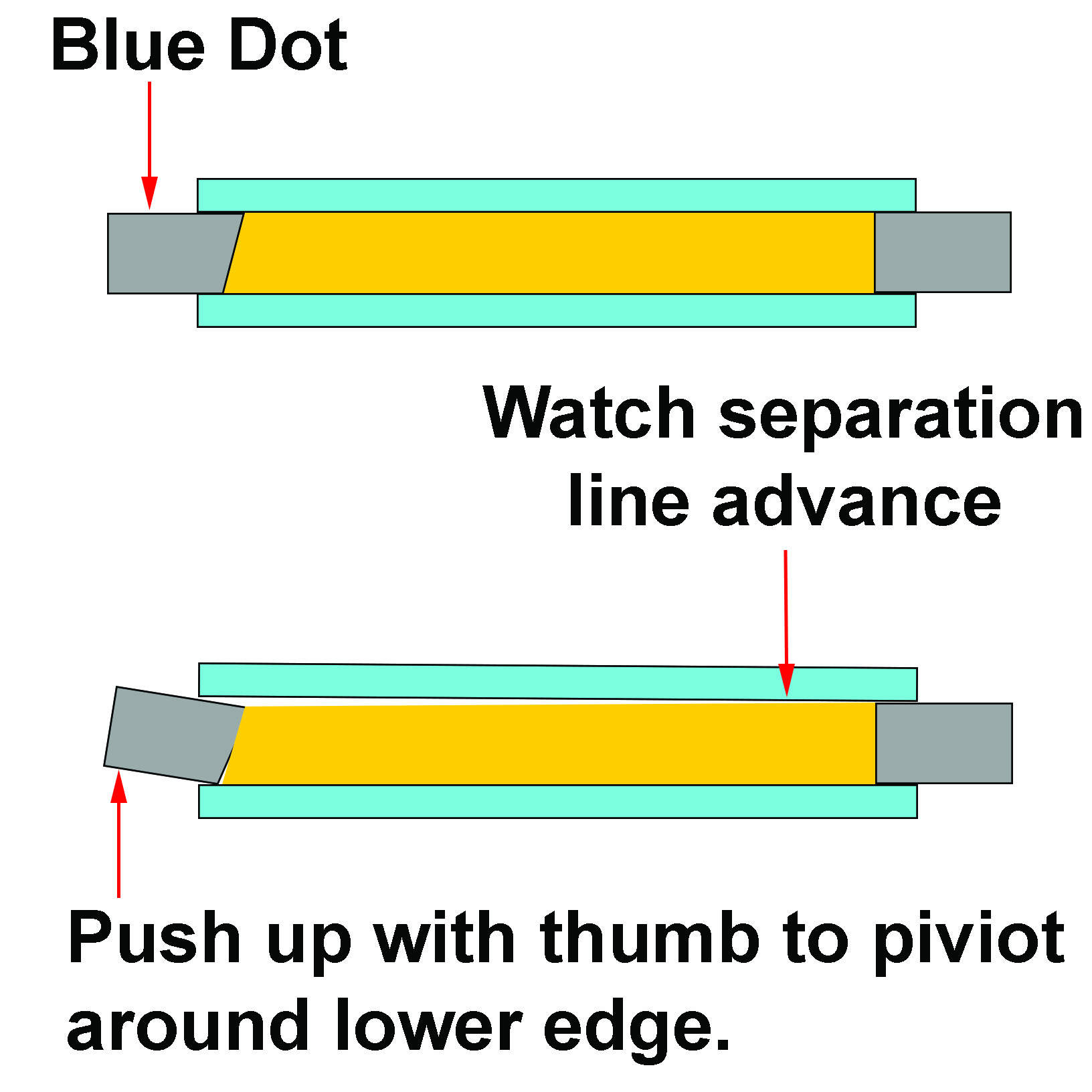** | **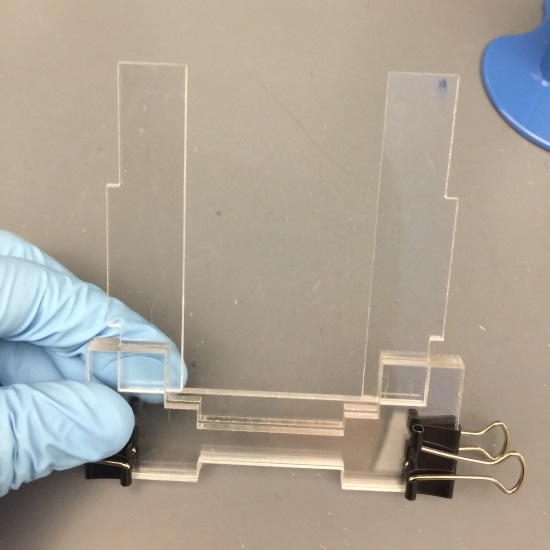** |
| --- | --- |
|  | **The should be marked so that the angled curf will be as shown on the right with the mold is placed on the bench top.** |

**When assembling the mold care must be taken to make sure the long spacers are placed in the mold with the correct orientation. This is because as the laser cuts the acrylic it tends to cut as a cone leaving the part with slightly angled sides instead of exactly 90^o^ sides. Then when the mold is placed on the benchtop to release the glass from the agar, the edge of spacer should be an acute angle with the agar. The acute angle helps compress the edge of the agar away from the upper piece of glass as the outside edge of the mold spacer is pivoted about the lower edge (see diagram above). The result is a more consistent separation of the agar from the top piece of glass.**

**Mold release agents like oils or silicon sprays or even rainX should not be utilized because they will contaminate the surface of the agar and possibly inhibit growth.**

**These methods take some practice to be consistent. An undergrad takes about 5-6 tries to get the hang of it.**

| **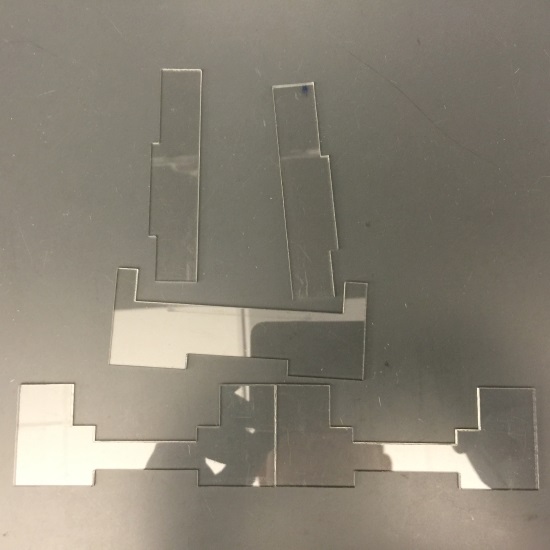** | 1. **Gather the 5 acrylic mold assembly.** 2. **Clean by spraying with 70% ethanol, wiping with kimwipe, and allowing to dry.** |
| --- | --- |
| **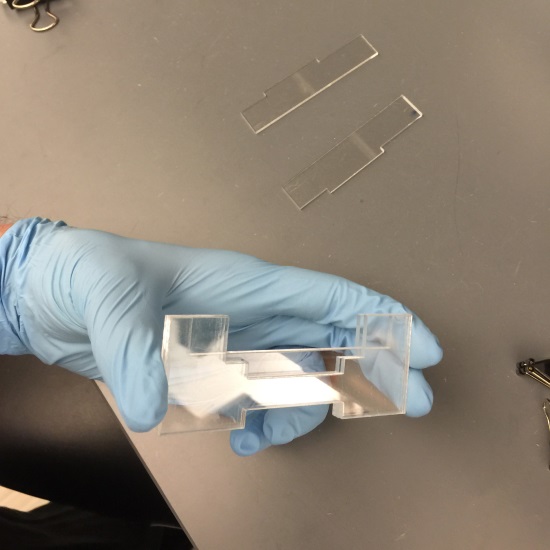** | 1. **Take three bottom pieces as shown and assemble so that the two identical pieces sandwitch the third piece.** |
| **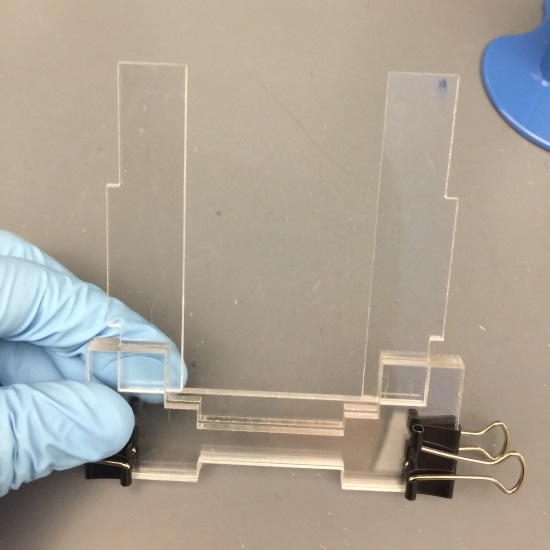** | 1. **Clamp the base pieces using a small binder clip** 2. **Then place uprights in making sure that the lazer curf is correctly positioned.** |

| **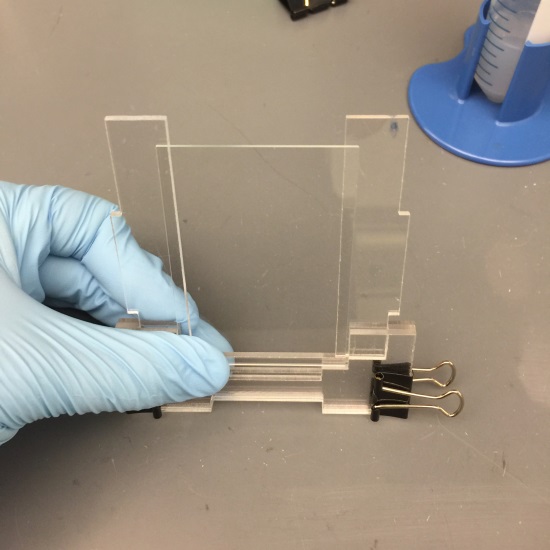** | 1. **Place cleaned and dried glass side on mold and hold** |
| --- | --- |
| **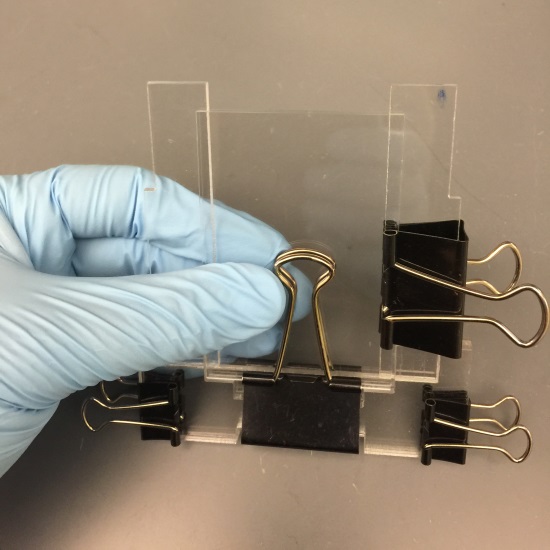** | 1. **Place glass slide on other side and clamp with larger binder clip.** 2. **Clamp both slides with larger binderclip** 3. **Make sure binder clip contacts above where slide overlaps the acrylic** |
| **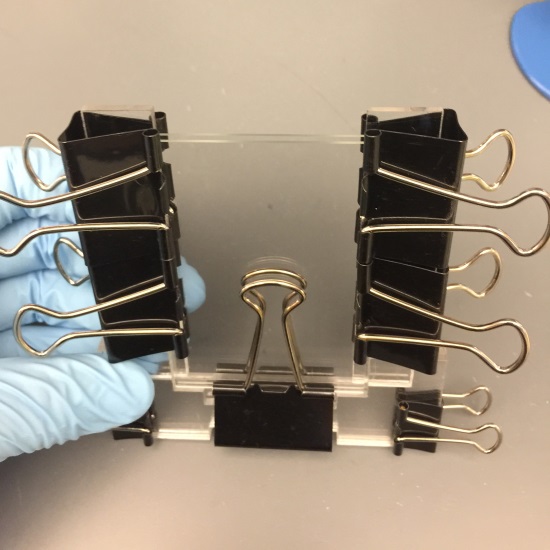** | 1. **Add Remaining binder clips. Again make sure the clamps contact the slide where it overlaps the acrylic. Once assembled, melt the agar accordingly.** 2. **When filling make sure to seal the edges by pipetting ~70μL: of media along inner side of the mold. This agar will quickly solidify and plug any leaks.** 3. **After filling allow the mold to cool for 50minutes to 1 hour at about 23C ambient temperature. If allowed to cool too much the agar may fracture in the mold.** |

**Mold Separation**

| **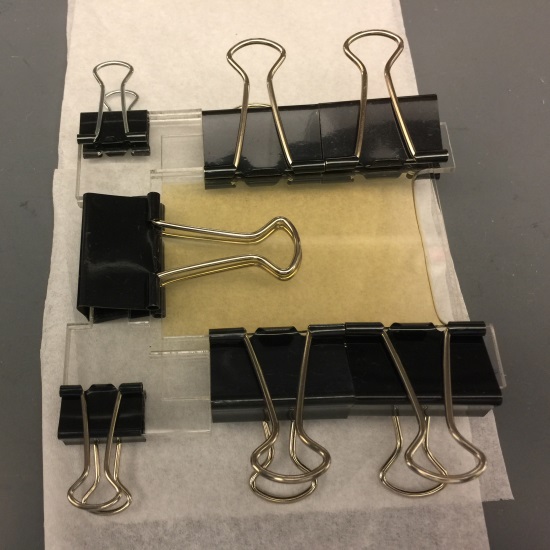** | 1. **After allowing the agar to cool for ~1 hr place mold on bench top.** |
| --- | --- |
| **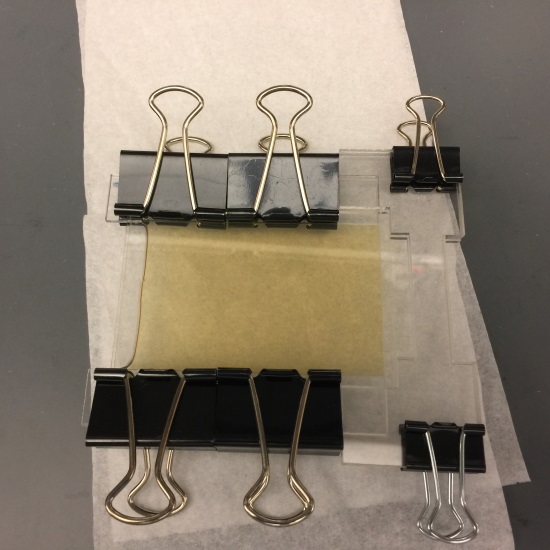** | 1. **Start by removing the bottom binder clip** |
| **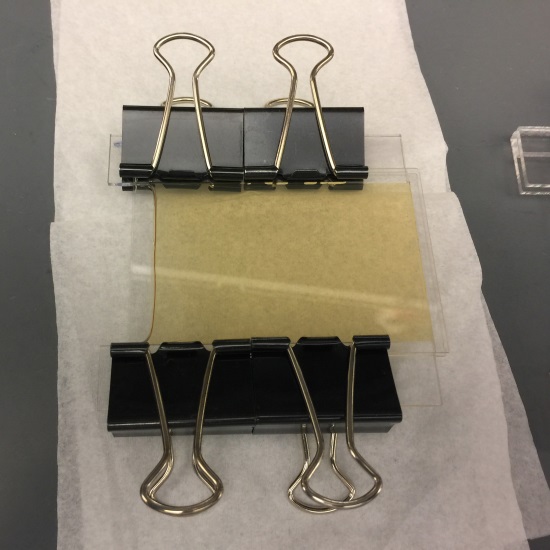** | 1. **The remove the bottom part of the mold.** |

| **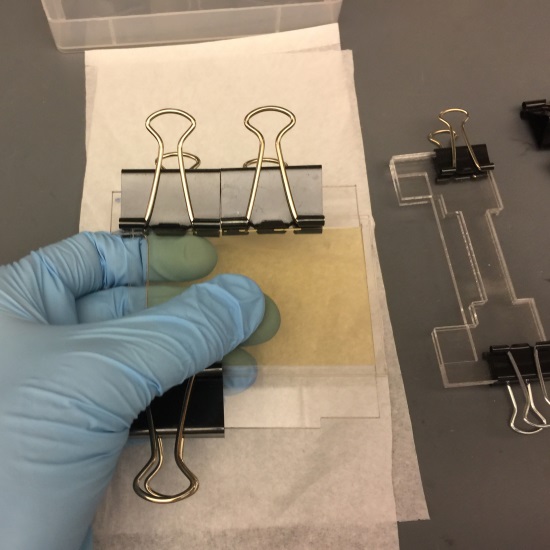** | 1. **Hold the slides by compressing them and then remove the binder clips. Be sure not to bump the model sides as you remove the binder clips. This will deform the media.** |
| --- | --- |
| **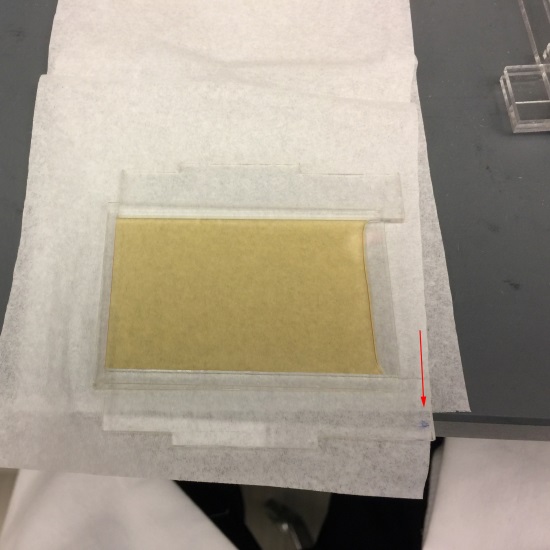** | 1. **Place mold on edge of bench top with the blue dot side of the mold facing up and in the lower left corner. This ensures the slightly acute edge of the mold is angled correctly for release. Please review the diagram below.** 2. **Place your thumbs underneath the acrylic and your first fingers on the top towards the inner edge of the mold.** 3. **Slowly and very carefully push up with your thumbs against mold as if pivoting the mold spacer about it’s lower edge.** 4. **Do so with constant but slowly increasing pressure and you should see a break and air bubble appear along the line where the top glass covers the mold spacer (see arrow)** |
| **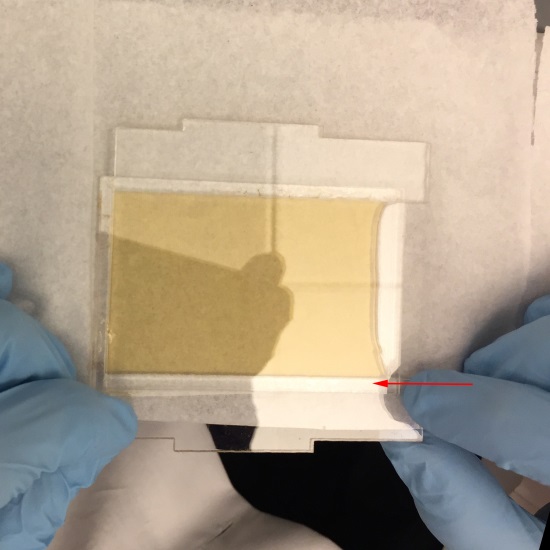** | **Side View**  **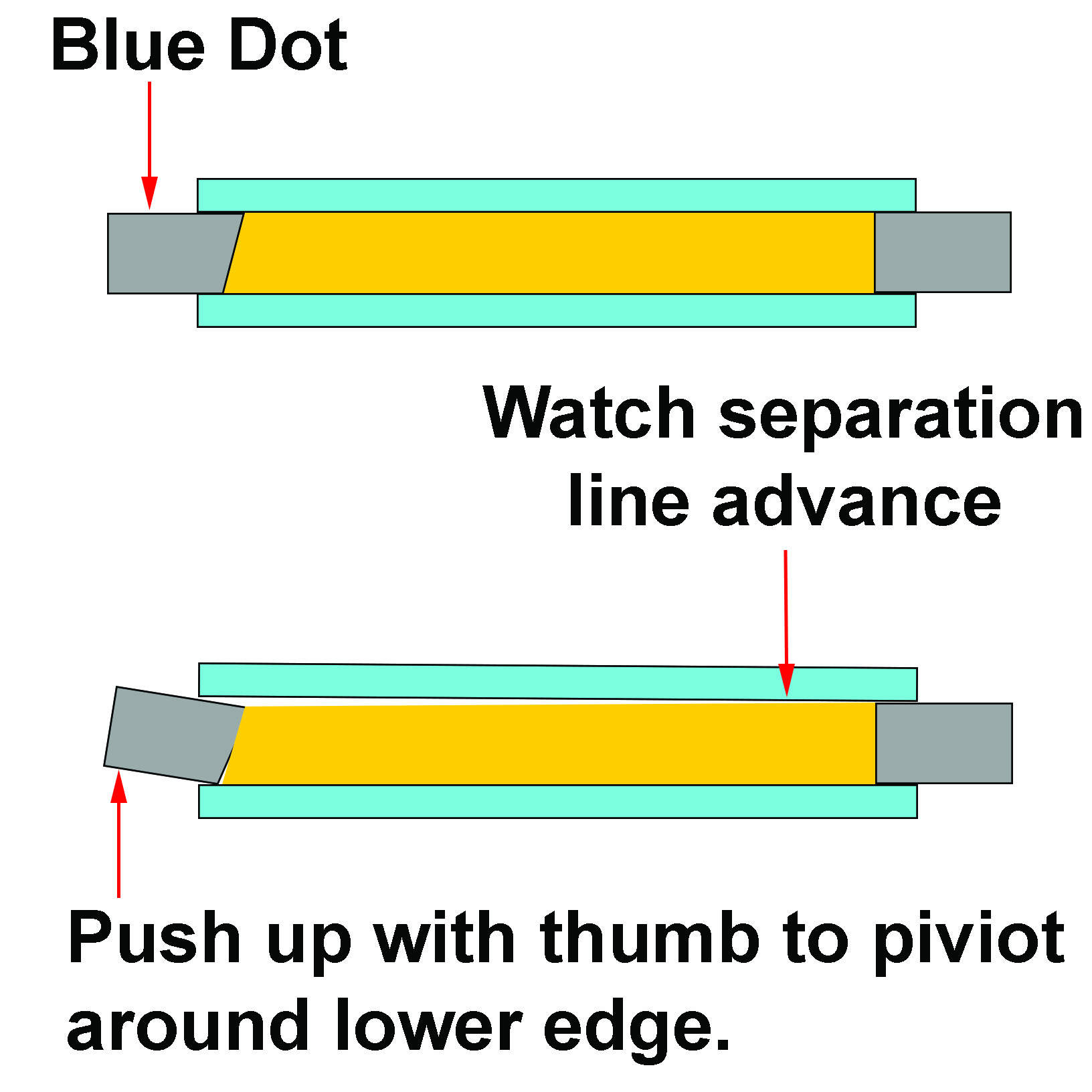** |

| **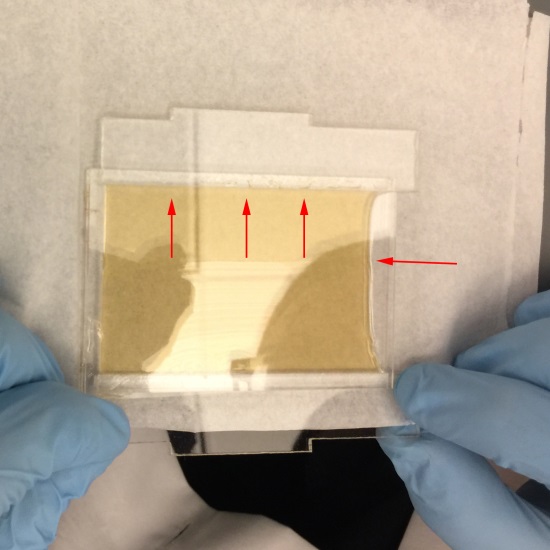** | 1. **After seeing the initial break line continue pushing up with constant pressure. The agar should start breaking away from the glass at this point.** 2. **Continue to pivot the mold upwards. This will lift the glass without sliding it. The break line(horizontal arrow) should continue to move away from you in the direction of the vertical arrows** 3. **At this point you should be able to see if the agar is sticking to both the bottom and top of the glass. This will occasionally occur with the left most edge. As long as the area deformed is not in the area where yeast is spotted this should be fine.** |
| --- | --- |
| **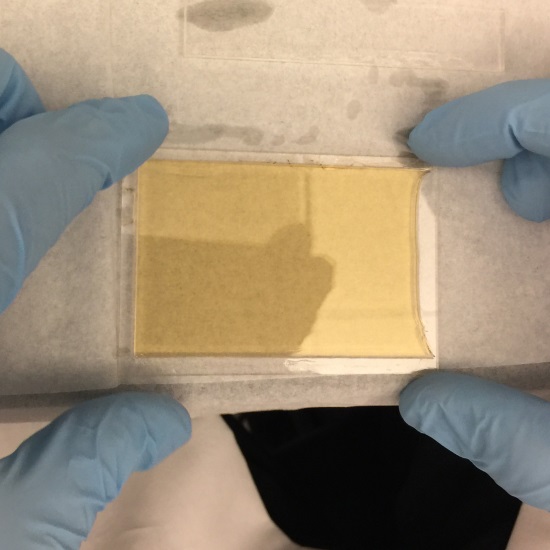** | 1. **As the glass comes completely free you should be able to grab it and lift it away.** 2. **Then remove the other mold piece using a similar motion to lift it free without moving the agar.** |
| **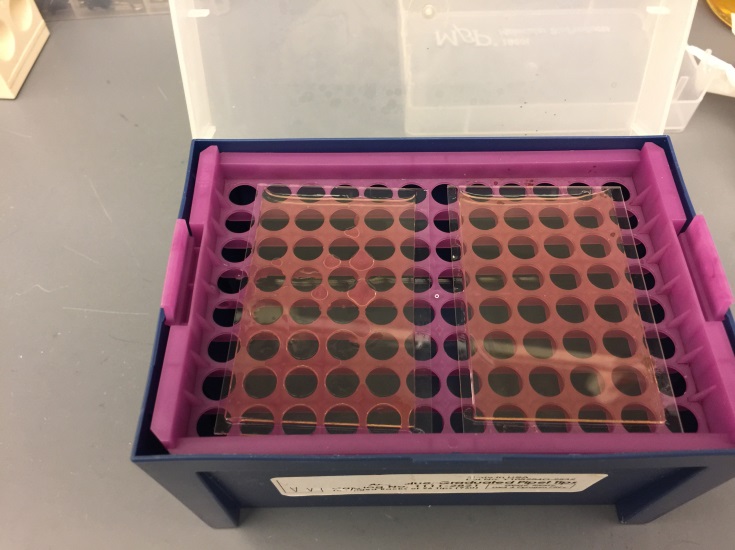** | 1. **Place the slides in a sterile tip box with some sterile high purity water in the bottom. Close the box and refrigerate over night for use the next day. If stored for longer the growth rates will become increasingly inconsistent.** |

**Preculture plate over night**

**Pre-Setup**

Obtain the following items

- 1x trough for multichannel pippet
- 1x 96 well plate flat bottom
- 1x Media
- 1x Box of tips (filter tips preferred)

**Protocol Steps:**

1. Seed culture into 200μL media
2. Grow over night

**ODELAY 1^st^ Diltuion plate over night**

**Pre-Setup**

+2Hrs Activate microscopes and incubation chambers

+2Hrs Set up folders on microscopes

+2Hrs take gel box out to equilibrate cast gel

+10min put ice and water in sonicator

Obtain the following items

- 1x troff for multichannel pippet
- 8 x 15mL Falcon tubes
- 7x 96 well plate flat bottom
- 1x Media
- 1x Spoting media without carbon source

**Protocol Steps:**

1. Dilute overnight culture 1:11 in fresh plate
   1. In Fresh plate add 20μL of overnight culture into 200 μL of media for 220μL total
2. Measure OD600of each culture in Synergy plate reader

| Start synergy program: | 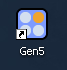 |
| --- | --- |
| Hit the Experiment under create new | 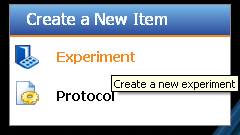 |
| Choose ODELAYDilution.exp and run the experiment | 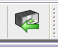 |
| Enter experiment name ODELAY “Date” “Time” “Experiment Name” iteration |  |
| Click on statistics tab and then click on Excel icon | 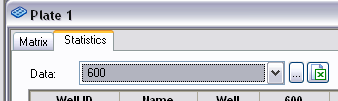 |
| Save Data to thumb drive and transfer thumb drive to Hamilton computer |  |

1. Dilute plate using Hamilton automated dilution.
   1. Open Hamilton Layout and Method Editor
   2. Run ODELAYDiltion_v1.med
   3. Run MATLAB Executable Convert SynergyFiles
   4. Enter 0.09 for target OD
   5. Click on Glu Correction and Gal Correction to 0.05 ( if unsuremeasure blank media in a identical plate)
   6. Click Generate File
   7. Select file in Hamilton method editor
   8. Click on stoplight icon to start method
   9. MAKE SURE TUBES ARE UNCAPPED AND PLATES HAVE LIDS REMOVED AND DUST COVERS ARE REMOVED FROM TIPS
   10. Load plates and tubes and tips on Hamilton Deck
   11. Click play
   12. Set number of 50μL tips
   13. Set number of 300μL tips
   14. Wait ~12 minutes for process to run
2. Culture Plate 5-6 hours at 30C
3. At this time:
   1. take agar plates from cooler
   2. Set up tips next to hydra
   3. Put fresh water and Ice in ultrasonic bath
4. Recover Diluted plate from Hamilton and Cover tips and recap falcon tubes
5. Cover with metal freezer seal (#)
6. Sonicate in ice bath for 30 seconds with plate floating
7. 150μL transfer sonicated culture to flat bottom plate
8. Spot on Hydra using appropriate hydra program

**Pre-Setup**

+2Hrs Activate microscopes and incubation chambers

+2Hrs Set up folders on microscopes

+10min put ice and water in sonicator

**Protocol Steps:**

**For Carbon Source Switch:**

1. Place plate in centrifuge
2. Spin 4000xg for 2 minutes
3. Carefully remove 180μL of media
4. Resuspend in 200μL of media without carbon source
5. OD culture in Synergy plate reader

| Start synergy program: | 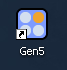 |
| --- | --- |
| Hit the Experiment under create new | 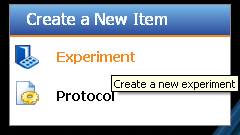 |
| Choose ODELAYDilution.exp and run the experiment | 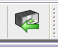 |
| Enter experiment name ODELAY “Date” “Time” “Experiment Name” iteration |  |
| Click on statistics tab and then click on Excel icon | 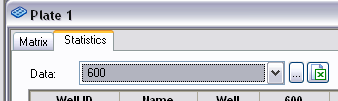 |
| Save Data to thumb drive and transfer thumb drive to Hamilton computer |  |

1. Dilute plate using Hamilton automated dilution.
   1. Open Hamilton Layout and Method Editor
   2. Run ODELAYDiltion_v1.med
   3. Run MATLAB Executable Convert SynergyFiles
   4. Enter 0.09 for target OD
   5. Click on Glu Correction and Gal Correction to 0.05 ( if unsuremeasure blank media in a identical plate)
   6. Click Generate File
   7. Select file in Hamilton method editor
   8. Click on stoplight icon to start method
   9. MAKE SURE TUBES ARE UNCAPPED AND PLATES HAVE LIDS REMOVED AND DUST COVERS ARE REMOVED FROM TIPS
   10. Load plates and tubes and tips on Hamilton Deck
   11. Click play
   12. Set number of 50μL tips
   13. Set number of 300μL tips
   14. Wait ~12 minutes for process to run
2. At this time:
   1. take agar plates from cooler
   2. Set up tips next to hydra
   3. Put fresh water and Ice in ultrasonic bath
3. Recover Diluted plate from Hamilton and Cover tips and recap falcon tubes
4. Cover with metal freezer seal (#)
5. Sonicate in ice bath for 30 seconds with plate floating
6. 150μL transfer sonicated culture to flat bottom plate
7. Spot on Hydra using appropriate hydra program

**Pre-Setup**

+2Hrs Activate microscopes and incubation chambers

+2Hrs Set up folders on microscopes

+10min put ice and water in sonicator

**Protocol Steps:**

**Without Carbon Source Switch:**

1. Resuspend cultures in plate (1 box of filter tips)
2. OD culture in Synergy plate reader

| Start synergy program: | 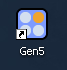 |
| --- | --- |
| Hit the Experiment under create new | 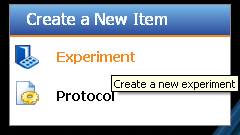 |
| Choose ODELAYDilution.exp and run the experiment | 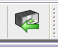 |
| Enter experiment name ODELAY “Date” “Time” “Experiment Name” iteration |  |
| Click on statistics tab and then click on Excel icon | 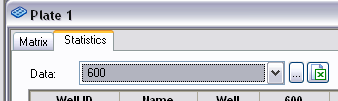 |
| Save Data to thumb drive and transfer thumb drive to Hamilton computer |  |

1. Dilute plate using Hamilton automated dilution.
   1. Open Hamilton Layout and Method Editor
   2. Run ODELAYDiltion_v1.med
   3. Run MATLAB Executable Convert SynergyFiles
   4. Enter 0.01 for target OD
   5. Set Glu Correction and Gal Correction to 0.05 ( if unsure measure blank media in an identical plate)
   6. Click Generate File
   7. Select file in Hamilton method editor
   8. Click on stoplight icon to start method
   9. MAKE SURE TUBES ARE UNCAPPED AND PLATES HAVE LIDS REMOVED AND DUST COVERS ARE REMOVED FROM TIPS
   10. Load plates and tubes and tips on Hamilton Deck
   11. Click play
   12. Set number of 50μL tips
   13. Set number of 300μL tips
   14. Wait ~12 minutes for process to run
2. At this time:
   1. take agar plates from cooler
   2. Set up tips next to hydra
   3. Put fresh water and Ice in ultrasonic bath
3. Recover Diluted plate from Hamilton and Cover tips and recap falcon tubes
4. Cover with metal freezer seal (#)
5. Sonicate in ice bath for 30 seconds with plate floating
6. 150μL transfer sonicated culture to flat bottom plate
7. Spot on Hydra using appropriate hydra program

| **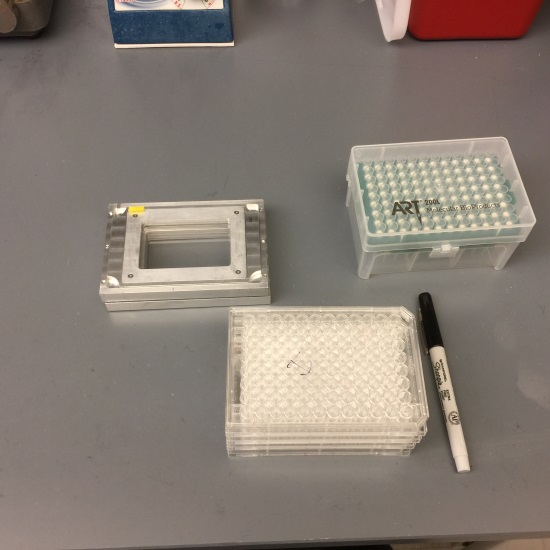** | 1. **Need 1x box of tips** 2. **4x 96 well plates** 3. **Mark plates with numbers 1 through 4 as these will be the quadrants from the source plate** |
| --- | --- |
| **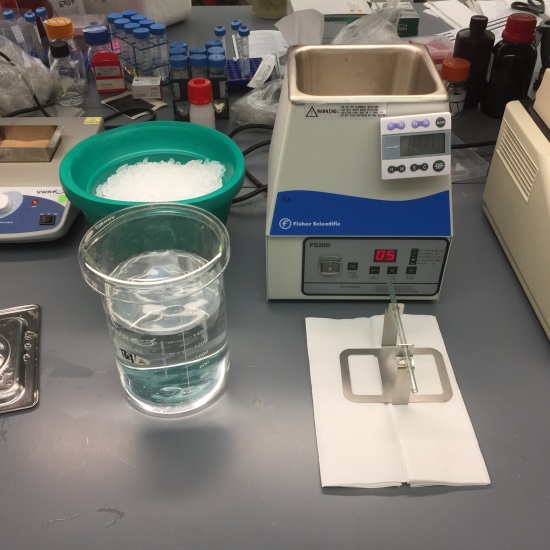** | 1. **Also get an ice bucket** 2. **Empty out any water remaining in the sonicator and replace with fresh diWater** 3. **Use a centrifuge 96 well adapter with a cut pippte.** |
| **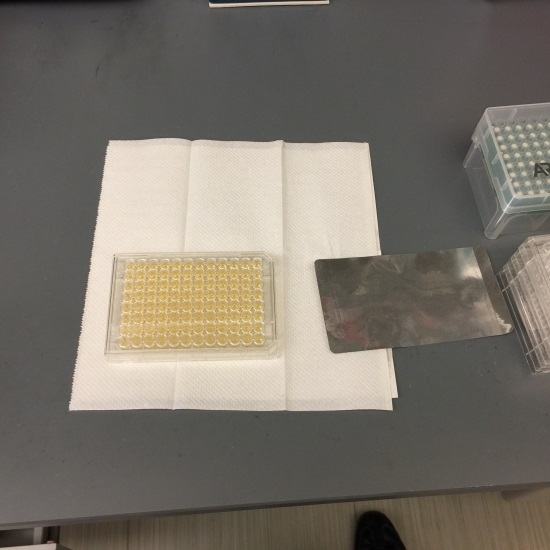** | 1. **And a Freezer aluminum film cover** |

| **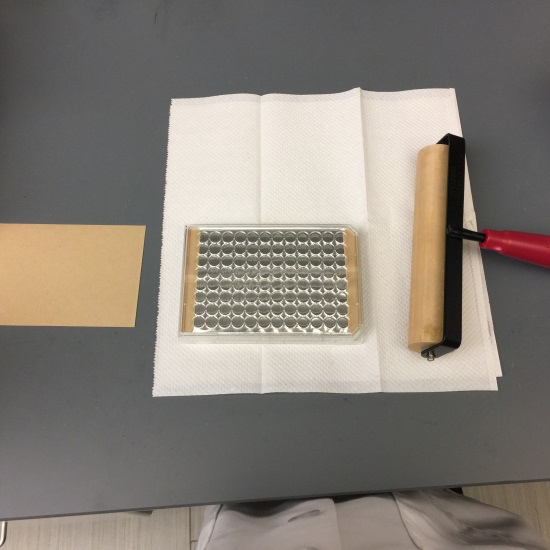** | 1. **Place the cover on the plate and seal it well** |
| --- | --- |
| **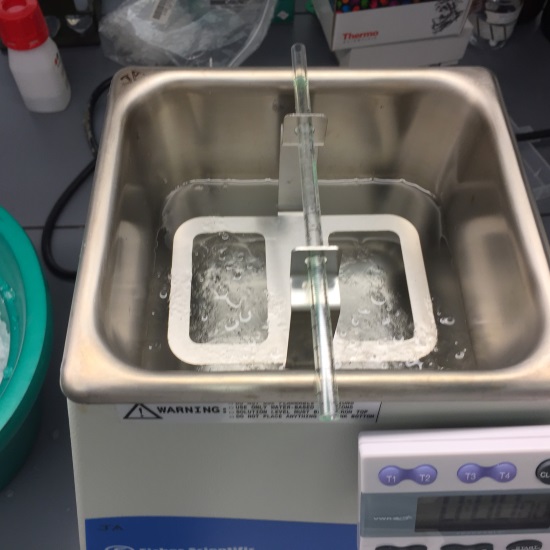** | 1. **Fill the sonicator with about 2-4 handfulls of ice and then add water until the water level is about 1cm above the centrifuge adapter** 2. **This will ensure the plate is just floating in the water and helps gauge putting a similar amount of water in the ultrasonic bath each time** |
| **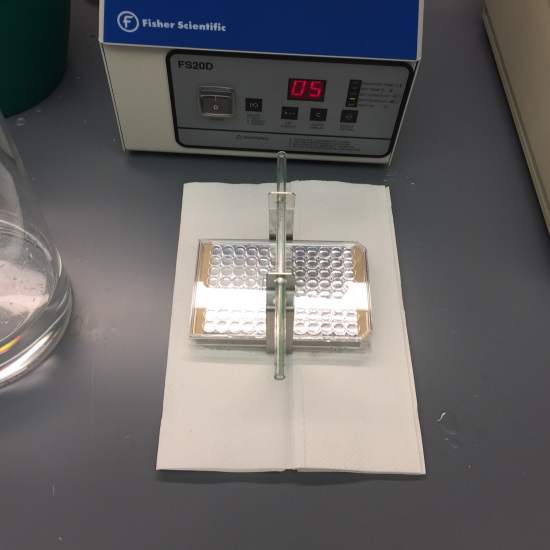** | 1. **Put the cover back on the plate and place the plate in the ultrasonic bath** |

| **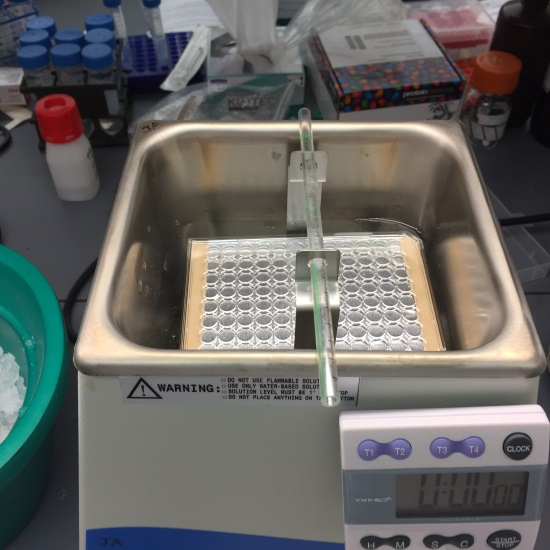** | 1. **Sonicate the plate for 30 seconds to breakup yeast mother-daughter yeast cells** |
| --- | --- |
| **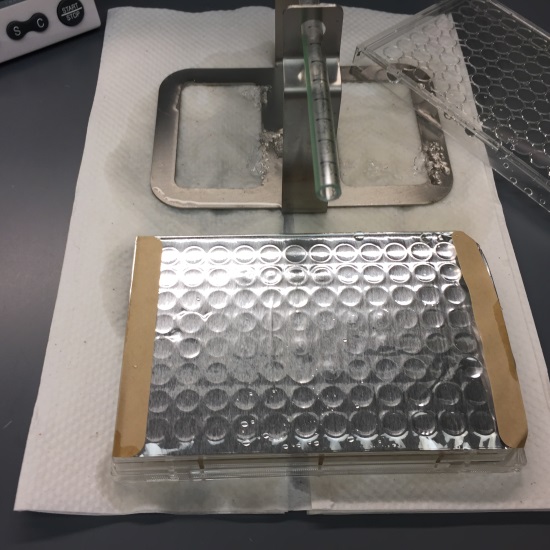** | 1. **Remove plate from ultrasonic bath and dry the plate as well as possible** |
| **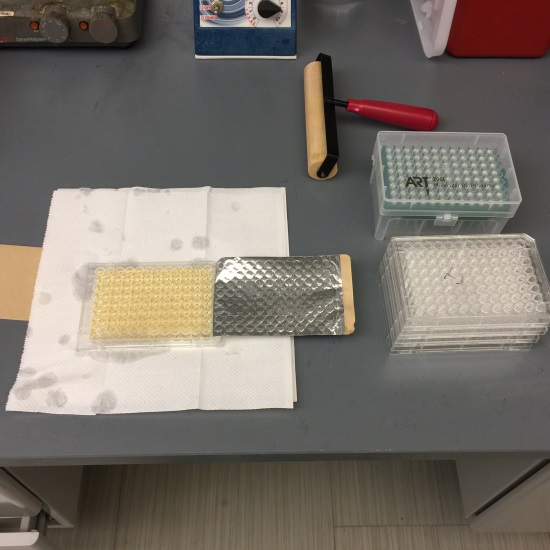** | 1. **Remove cover and film carefully without causing cross-contamination** |

| **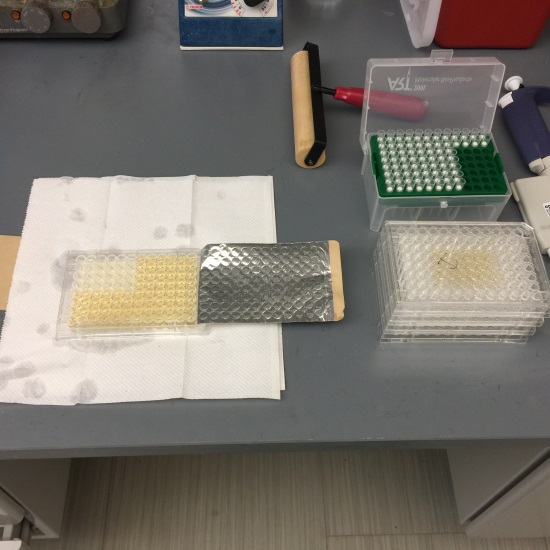** | 1. **Now fill the remaining plates. The upper left quadrant into the center 24 wells of plate 1** |
| --- | --- |
| **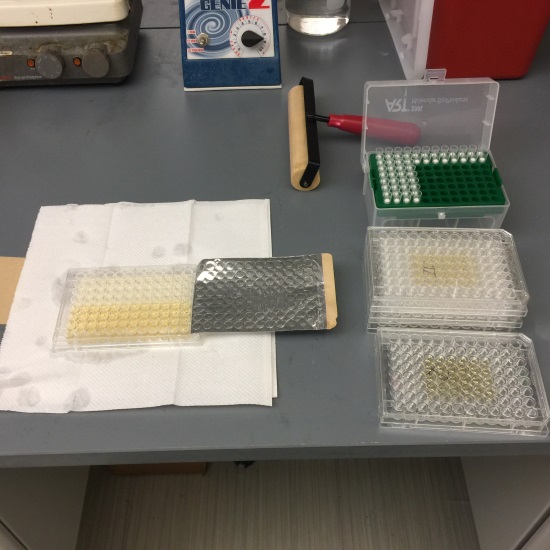** | 1. **Now fill the remaining plates. The upper right quadrant into the center 24 wells of plate 2** |
| **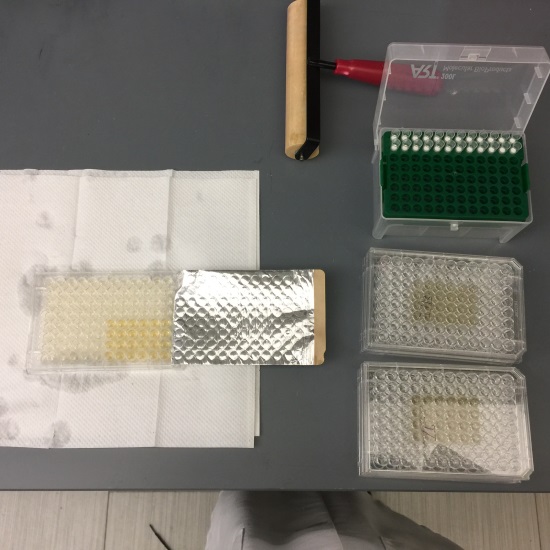** | 1. **Now fill the remaining plates. The Lower Left quadrant into the center 24 wells of plate 3** |

| **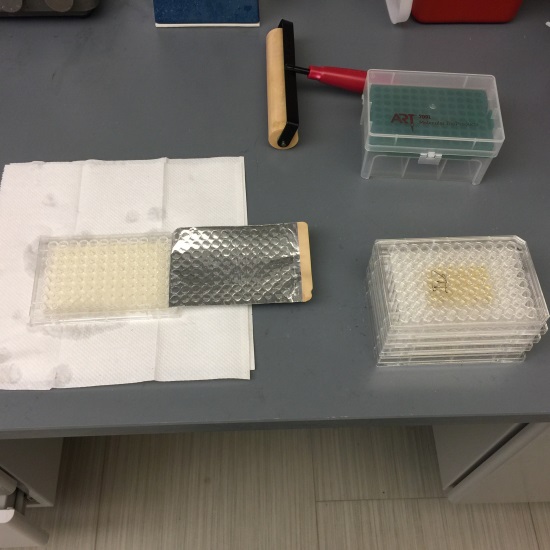** | 1. **Now fill the remaining plates. The Lower Right quadrant into the center 24 wells of plate 4** |
| --- | --- |
|  |  |
|  |  |

| 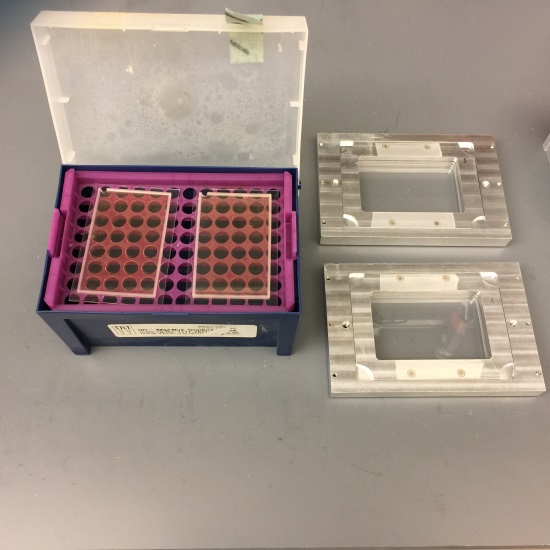 | 1. **Remove agar plates stored at 4^o^C over night** |
| --- | --- |
| 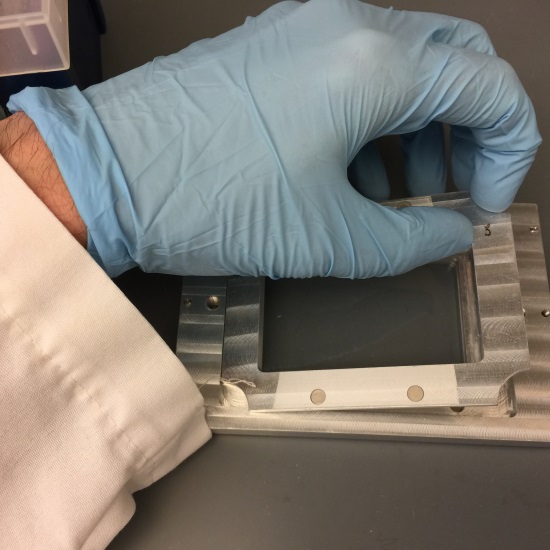 | 1. **Take slide clamp off of mount** |
| 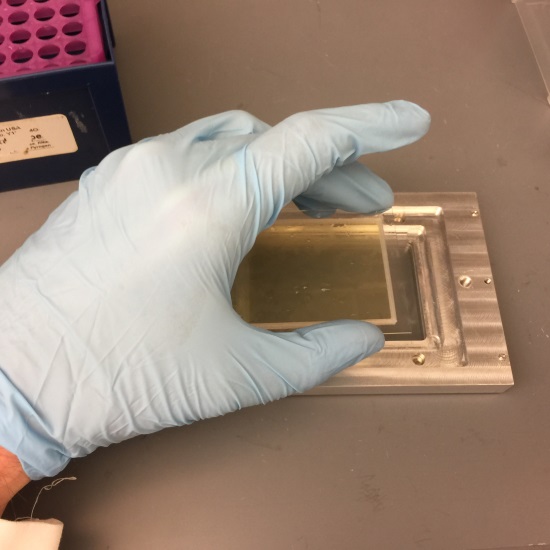 | 1. **Carefull plate agar plate into recessed area of the stage chamber mount.** |

| 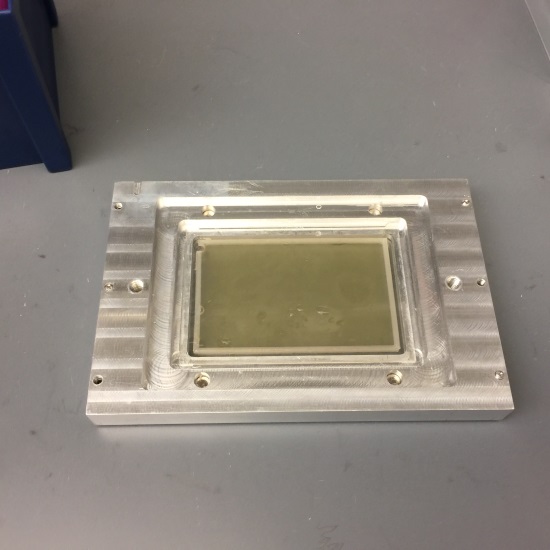 | 1. **Make sure the orientation of the slide is consistent from experiment to experiment.** |
| --- | --- |
| 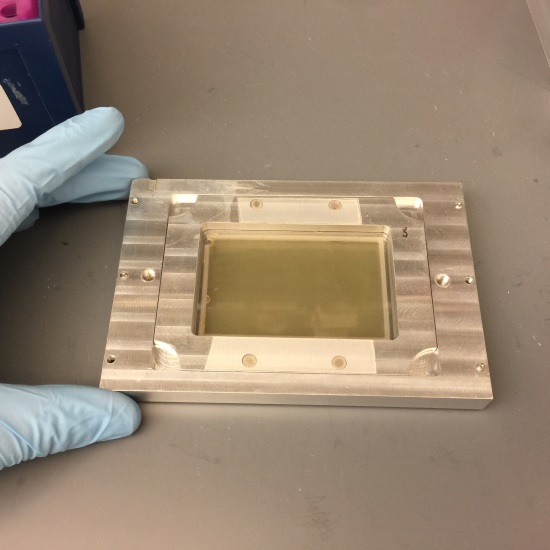 | 1. **Check to make sure the clamp is flush with the bottom of the chamber. If it is crooked then the slide may not be fully in the recess.** |
|  | **Note: These show a new method of producing slide using a 3D printed gasket. Details on this method will be forthcoming.** |

**Spotting on Agar using Hydra**

1x box of hydra tips

Use ODLEAY

| 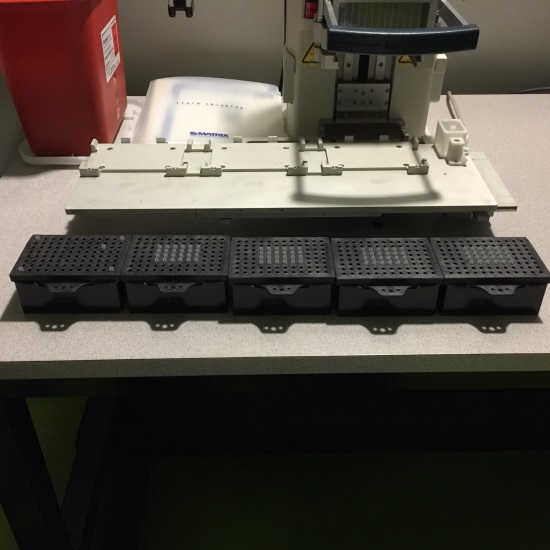 | 1. **Lay out the tips as shown. The box on the far left will need to have one tip in the C10 position as well as four tips with their ends cut off in the A01, A12, H01, and H12 positions. The four cut off tips provide stability.** |
| --- | --- |
| 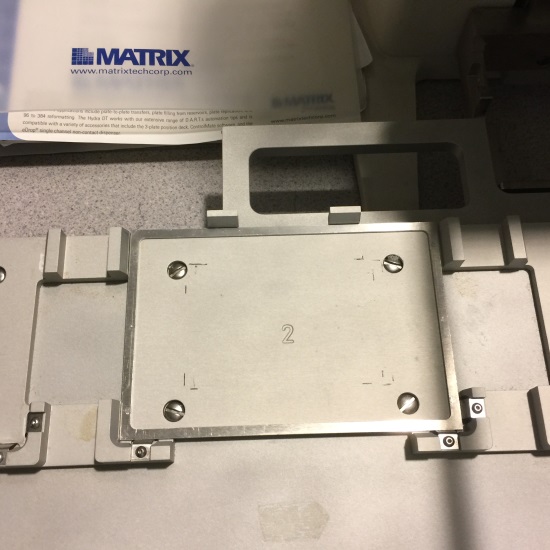 | 1. **Place the leveling spacer into the center plate position. This spacer provides contact for the leveling screws so that the slide chamber can be leveled with the tips.** |
| 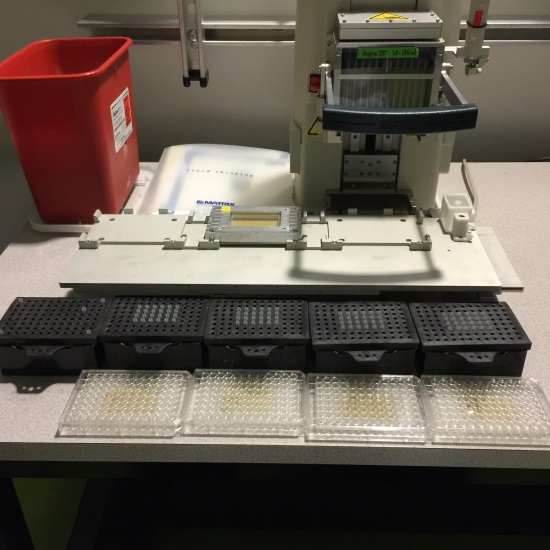 | 1. **Place the plates on the table inorder of their quadrant. 1 on the left and four all the way on the right.** |

| 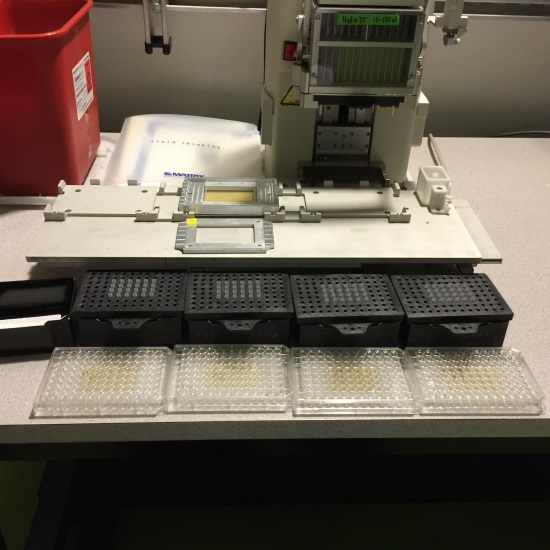 | 1. **Remove the top cover from the slide chamber mount.** 2. **Place the first tip punch into the Hydra and start the spotting program** |
| --- | --- |
| 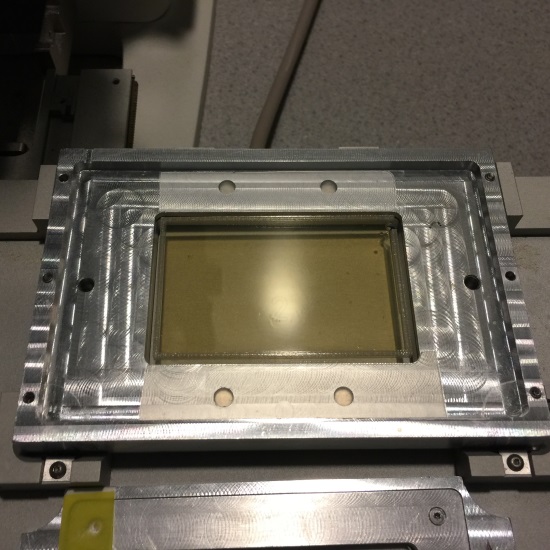 | 1. **This should punch a hole in the agar which will later be used for aligning the origin.** |
| 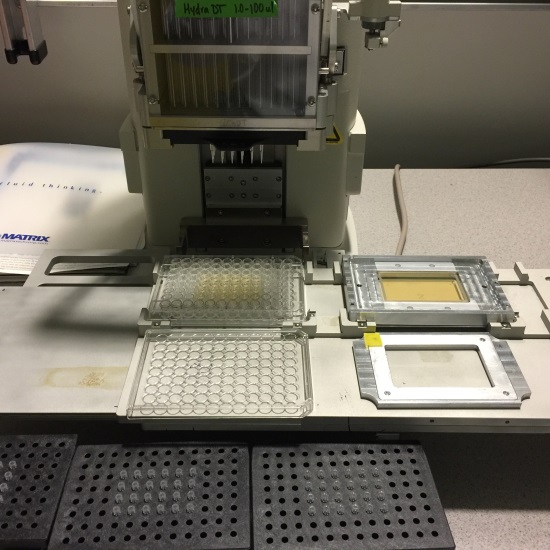 | 1. **Place plate 1 to spot the first quadrant. Make sure the lid is removed and continue the spotting program** |

| 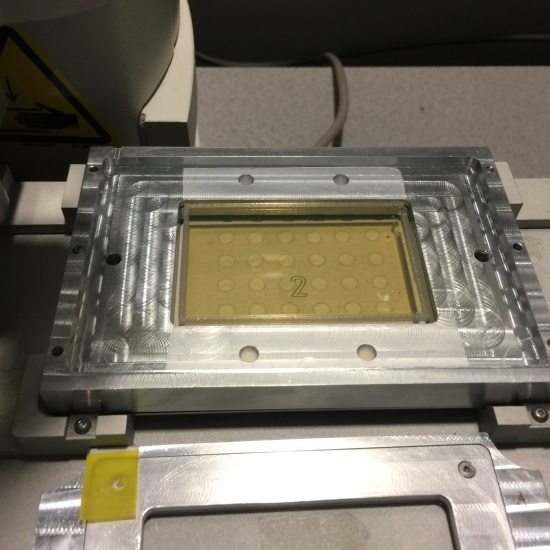 | 1. **Check to make sure all spots are present. Also wait for them to dry** 2. **When the spots are ~1mm in diameter it should be ok to start the next spot** |
| --- | --- |
| 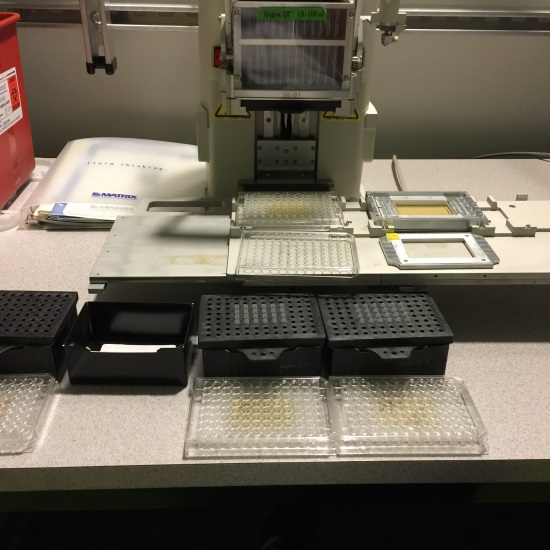 | 1. **Put the used tips in the biohazard container, and put in the fresh tips as well as swap out for the 2^nd^ quadrant plate** |
| 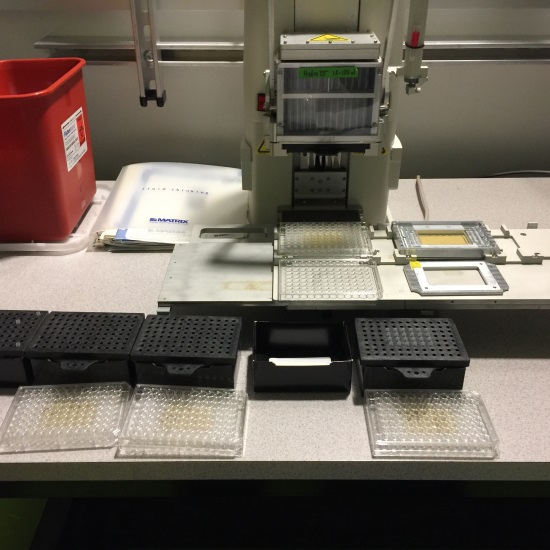 | 1. **Repeat for the third quadrant** |

| 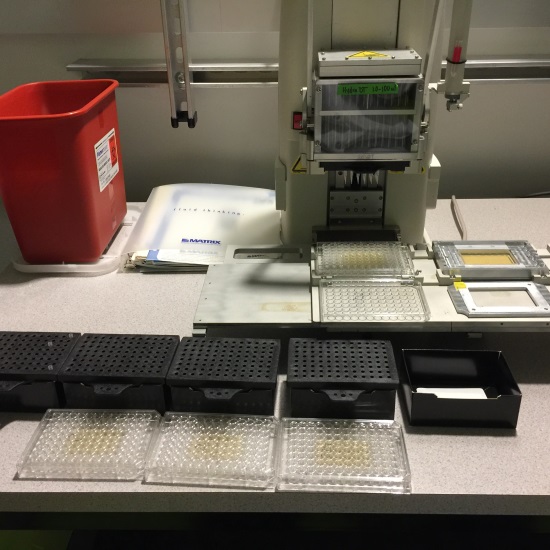 | 1. **And repeat again for the 4^th^ quadrant** |
| --- | --- |
| 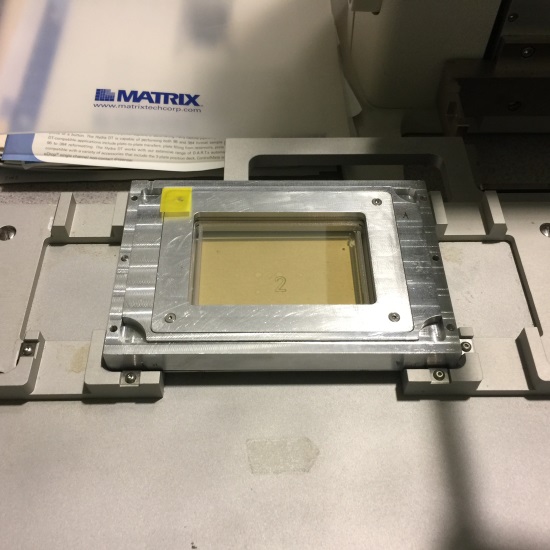 | 1. **When the spots are dry, replace the slide chamber cover and flip over.** |
| 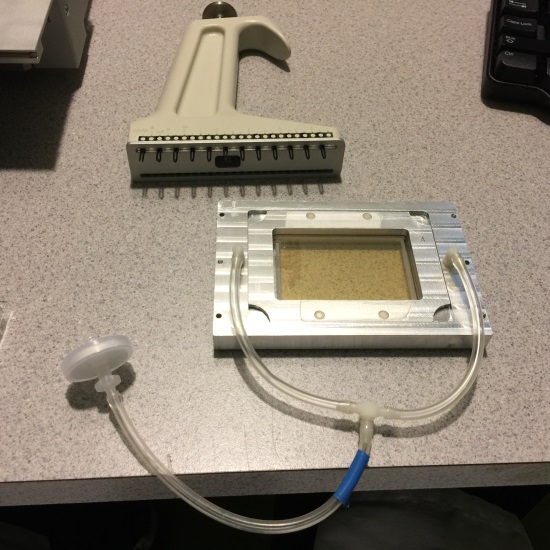 | 1. **Install the tubing connections as shown with an air filter.** |

| 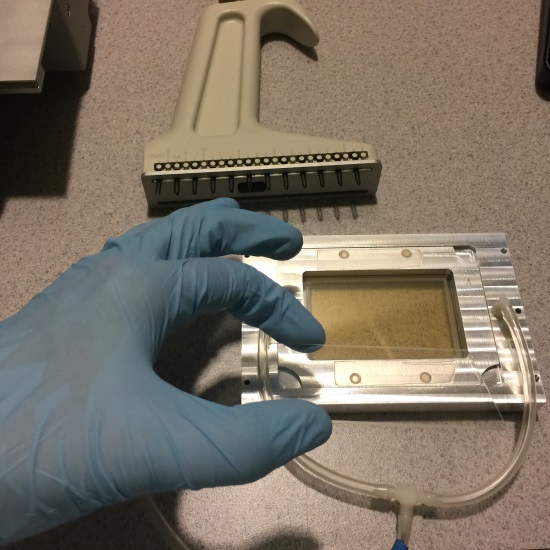 | 1. **Place a glass slide on the top side of the chamber. This slide is crucial for reducing the heat flux to the agar which then can drive condensation onto the objective coverslip side of the chamber. Future chamber designs will have mounts for this piece of glass.** |
| --- | --- |
| 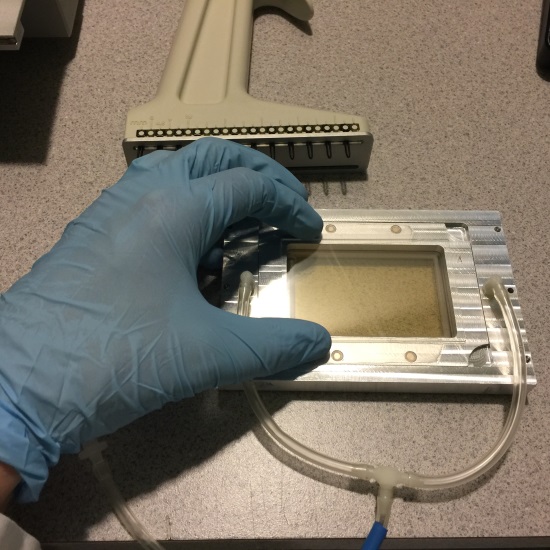 | 1. **Don’t forget this cover slip. Depending on the microscope configuration, This cover slip may or may not be needed. It prevents heating from the illumination side from causing condensation on the objective side.** |
|  |  |

| 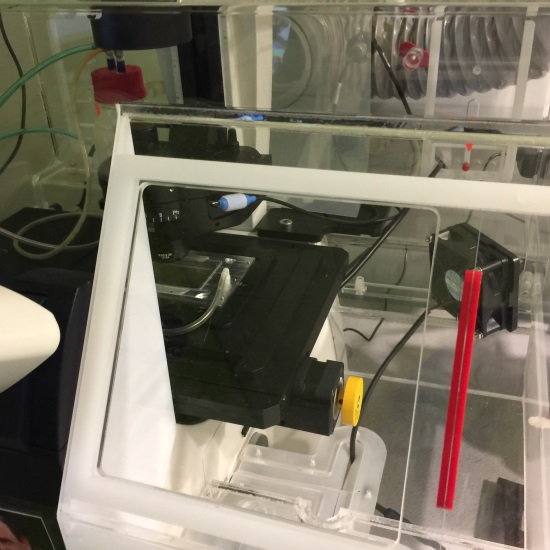 | 1. **This fan is important to blow hot air onto the objective side of the chamber. It prevents condensation from forming on the cover slip.** |
| --- | --- |
| 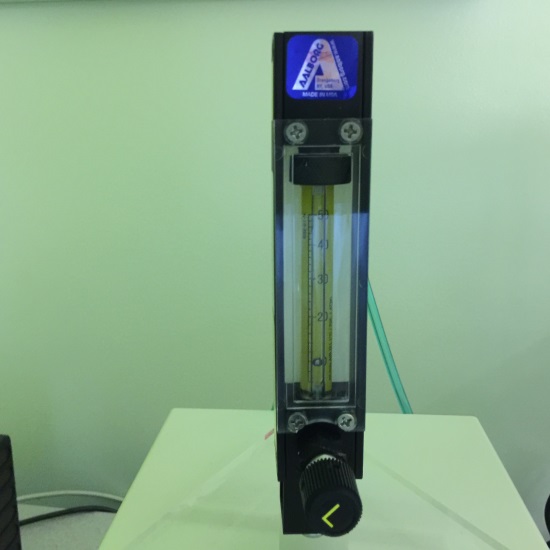 | 1. **Set the air flow rate through the bubbler to 10mL/min** |
| 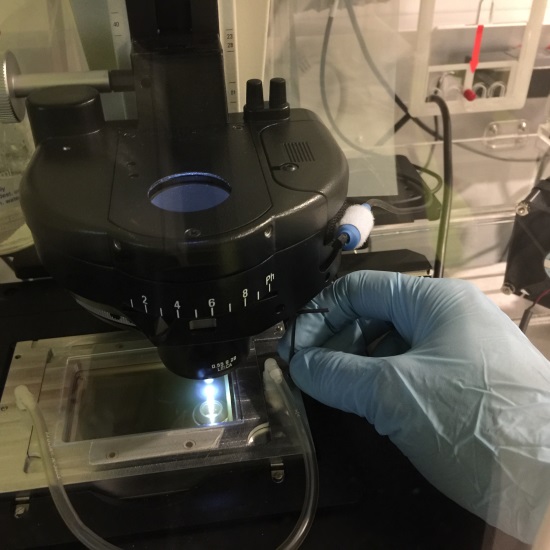 | 1. **Level stage by moving to positions closest to the screws and focusing the image using the allen key.** |
