# Supplementary material for: One-Cell Doubling Evaluation by Living Arrays of Yeast, ODELAY!
Source: G3 (Bethesda). 2016 Nov 16;7(1):279–88. doi: 10.1534/g3.116.037044 (PMC5217116; doi:10.1534/g3.116.037044)
Supplement: Supplementary file 2 [file 279FileS1.zip › ODELAY Hardware Design and Protocol/ODELAY Stage Mounts and Agar Mold Files/ODELAY stagemount v2p9 CoverSlip Clamp.pdf]

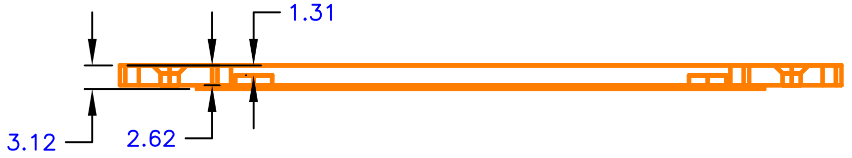

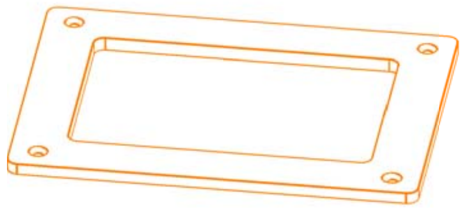

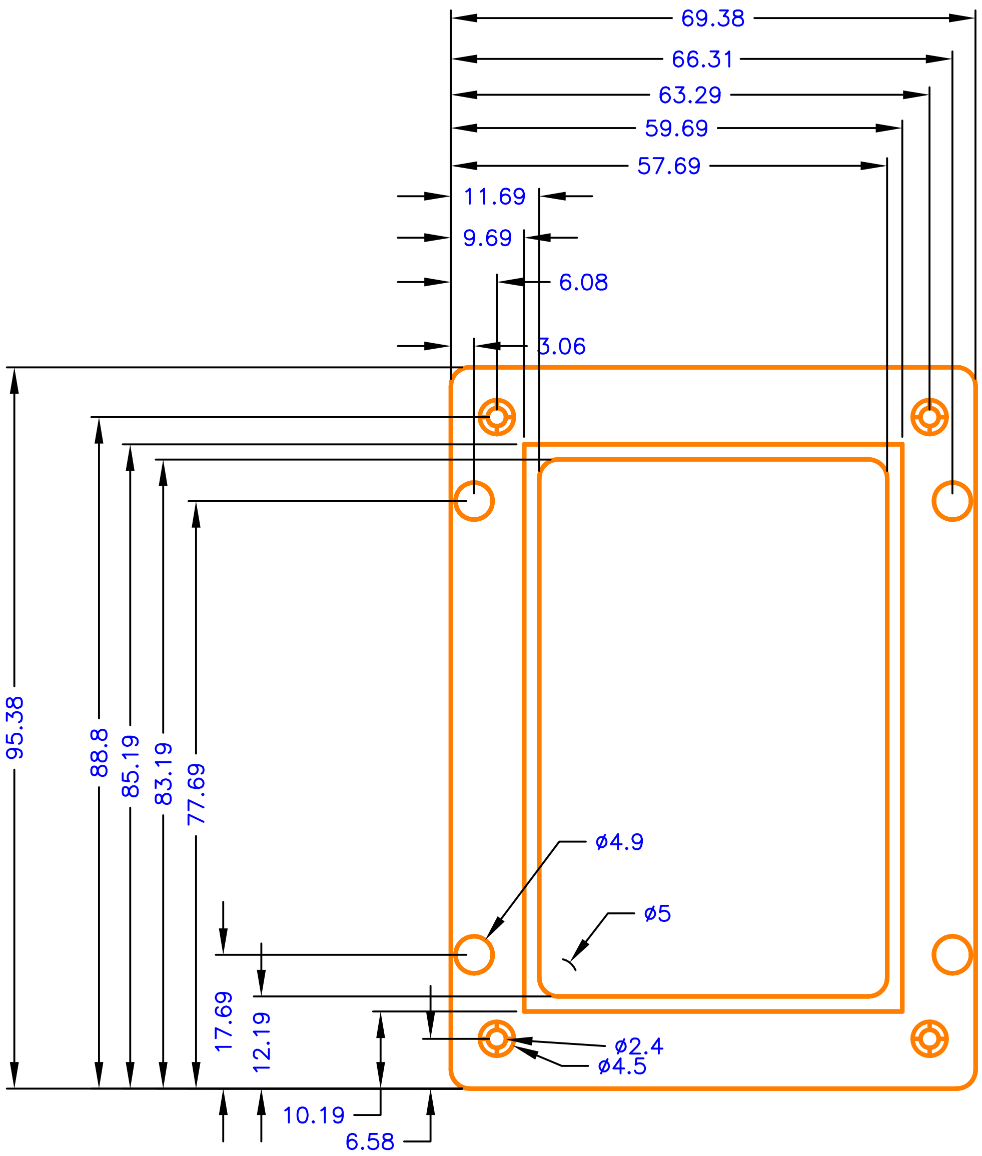

ODELAY CoverSlip Clamp	Thurston Herricks<br>Institute for Systems Biology	SCALE 1:1 (in millimeters)
ODELAY stagemount v2p9.dwg	Al 6061	2013-10-18
