# Supplementary figures and images for: One-Cell Doubling Evaluation by Living Arrays of Yeast, ODELAY!
Source: G3 (Bethesda). 2016 Nov 16;7(1):279–88. doi: 10.1534/g3.116.037044 (PMC5217116; doi:10.1534/g3.116.037044)

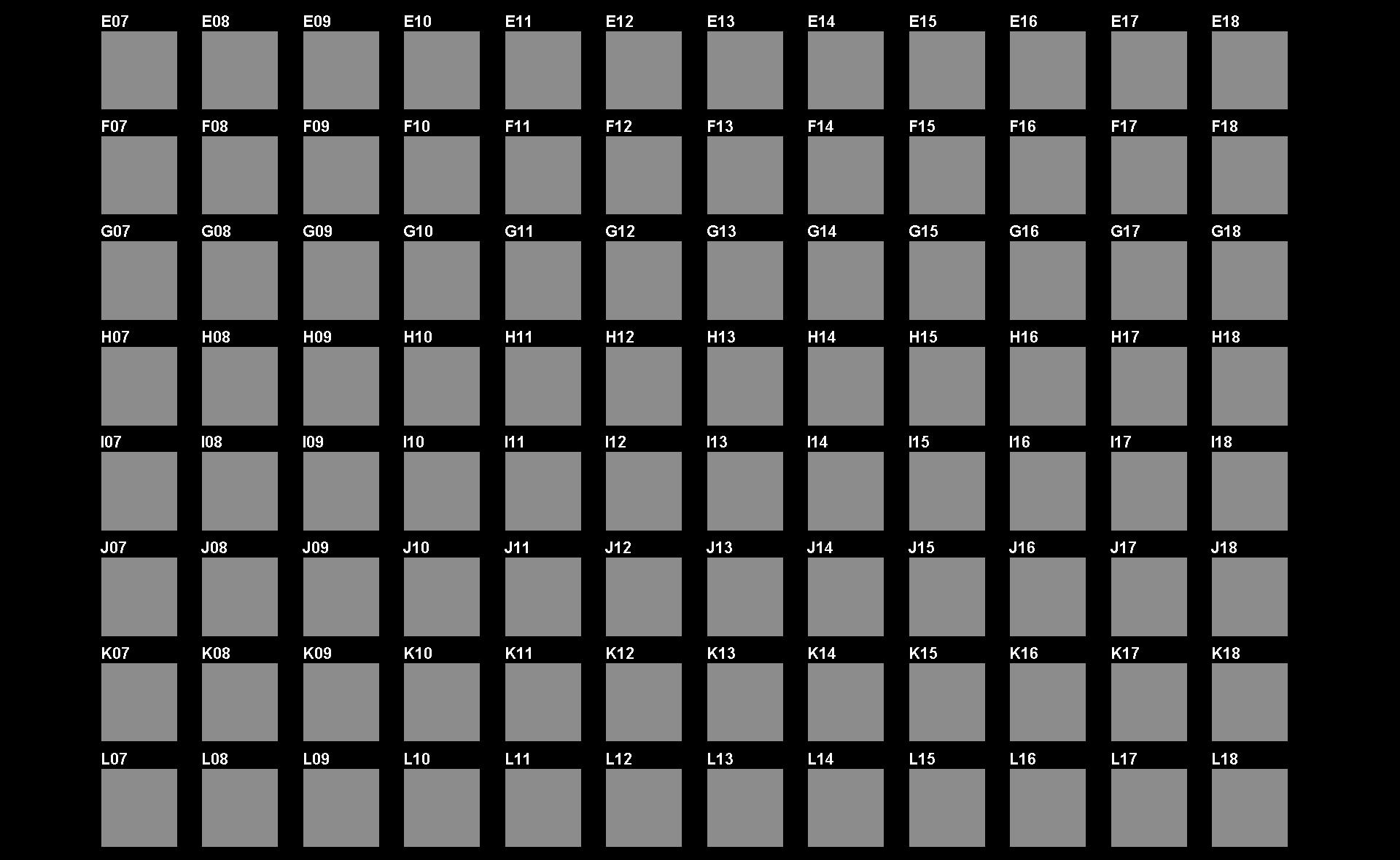

Supplement: Supplementary file 3 [file 279FileS2.zip › ODELAY Software and Example Dataset/ODELAY Image Processing Tool/ODELAYGUI.jpg]

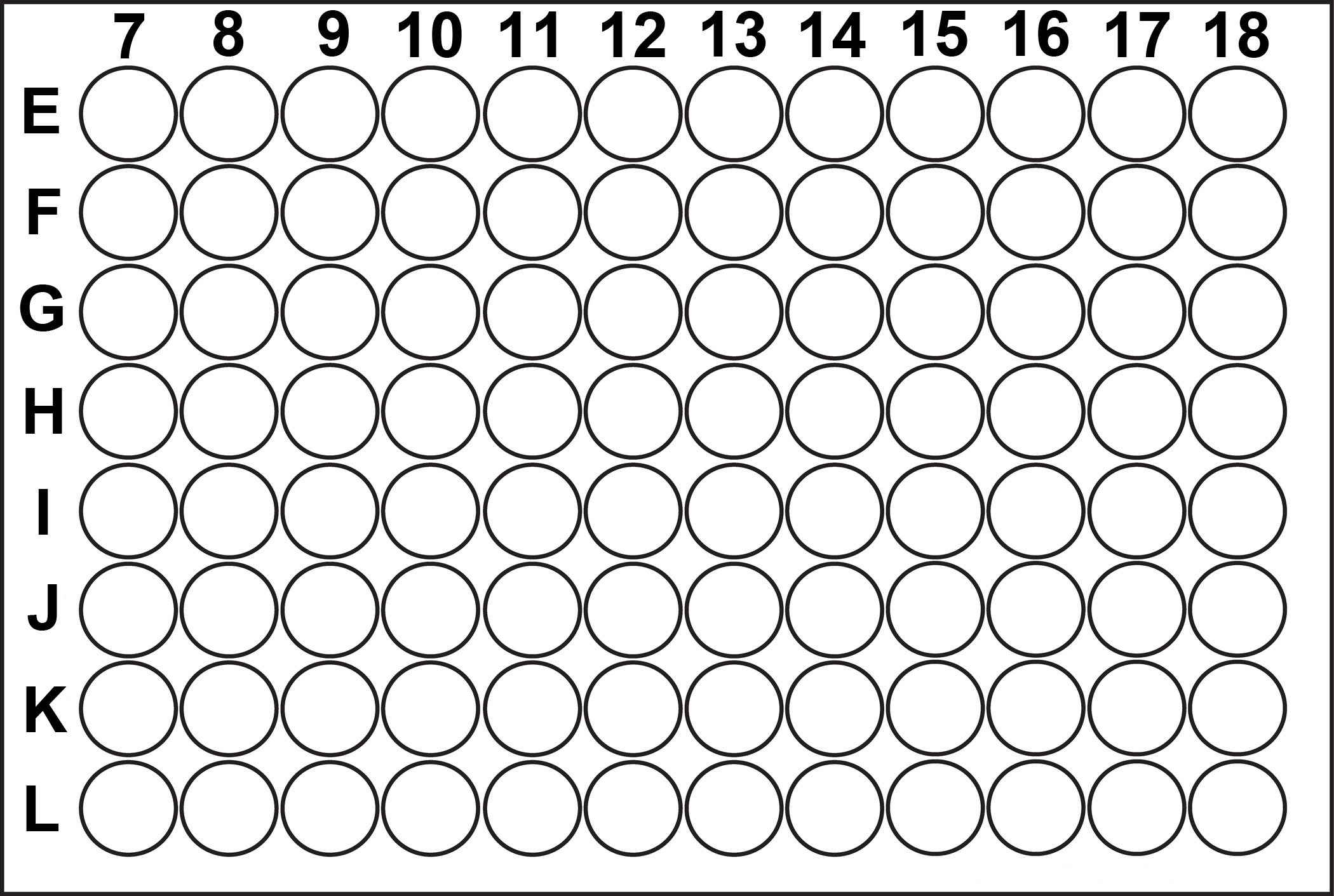

Supplement: Supplementary file 3 [file 279FileS2.zip › ODELAY Software and Example Dataset/ODELAY Microscope Control/96_well_plate_384_label.jpg]

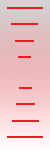

Supplement: Supplementary file 3 [file 279FileS2.zip › ODELAY Software and Example Dataset/ODELAY Microscope Control/Focus Control.tif]

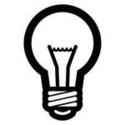

Supplement: Supplementary file 3 [file 279FileS2.zip › ODELAY Software and Example Dataset/ODELAY Microscope Control/light-bulb-clip-art-black-and-white-light-bulb-icon_small.jpg]

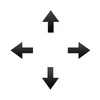

Supplement: Supplementary file 3 [file 279FileS2.zip › ODELAY Software and Example Dataset/ODELAY Microscope Control/Stage Arrows.tif]
