# Supplementary material for: One-Cell Doubling Evaluation by Living Arrays of Yeast, ODELAY!
Source: G3 (Bethesda). 2016 Nov 16;7(1):279–88. doi: 10.1534/g3.116.037044 (PMC5217116; doi:10.1534/g3.116.037044)
Supplement: Supplementary file 4 [file 279TableS1.pdf]

**Table S1: All strains investigated in this study.**

| Strain Common Name | Strain Systematic Name | Strain Common Name | Strain Systematic Name | Strain Common Name | Strain Systematic Name | Strain Common Name | Strain Systematic Name |
|---|---|---|---|---|---|---|---|
| Acs1 | YAL054C | Hst2 | YPL015C | Nup133 | YKR082W | Shg1 | YBR258C |
| Adr1 | YDR216W | Hst4 | YDR191W | Nup170 | YBL079W | Sif2 | YBR103W |
| Ahc1 | YOR023C | Htl1 | YCR020W-B | Nup188 | YML103C | Sin3 | YOL004W |
| Aim4 | YBR194W | Htz1 | YOL012C | Nup2 | YLR335W | Sir3 | YLR442C |
| Arp5 | YNL059C | Ies1 | YFL013C | Nup53 | YMR153W | Sir4 | YDR227W |
| Arp6 | YLR085C | Ies3 | YLR052W | NUP60 | YAR002W | Snf11 | YDR073W |
| Asf1 | YJL115W | Ioc2 | YLR095C | NUP84 | YDL116W | Snf12 | YNR023W |
| Bre1 | YDL074C | Ioc3 | YFR013W | Oaf3 | YKR064W | Snf2 | YOR290C |
| Bre2 | YLR015W | Ioc4 | YMR044W | Paf1 | YBR279W | Snf5 | YBR289W |
| Cdc73 | YLR418C | Isw1 | YBR245C | Pex3 | YDR329C | Snf6 | YHL025W |
| Chd1 | YER164W | Isw2 | YOR304W | Pho23 | YNL097C | Snl1 | YIL016W |
| Chz1 | YER030W | Itc1 | YGL133W | Pip2 | YOR363C | Snt1 | YCR033W |
| Dot1 | YDR440W | Kap114 | YGL241W | Pml39 | YML107C | Spp1 | YPL138C |
| Dyn2 | YDR424C | Kap120 | YPL125W | Pom152 | YMR129W | Spt20 | YOL148C |
| Eaf3 | YPR023C | Kap122 | YGL016W | Pom34 | YLR018C | Spt3 | YDR392W |
| Eaf5 | YEL018W | Kap123 | YER110C | Rad54 | YGL163C | Spt7 | YBR081C |
| Eaf6 | YJR082C | Leo1 | YOR123C | Rad6 | YGL058W | Sum1 | YDR310C |
| Eaf7 | YNL136W | Lge1 | YPL055C | Rco1 | YMR075W | Swc3 | YAL011W |
| Esc1 | YMR219W | Mad1 | YGL086W | Rpd3 | YNL330C | Swc5 | YBR231C |
| Fpr4 | YLR449W | Mad2 | YJL030W | Rph1 | YER169W | Swd1 | YAR003W |
| Gcn5 | YGR252W | Mak10 | YEL053C | Rsc2 | YLR357W | Swd3 | YBR175W |
| Gfd1 | YMR255W | Mak3 | YPR051W | Rtf1 | YGL244W | Swi3 | YJL176C |
| Gis1 | YDR096W | Mdm20 | YOL076W | Rxt2 | YBR095C | Swr1 | YDR334W |
| Gtt3 | YEL017W | Mip6 | YHR015W | Rxt3 | YDL076C | Taf14 | YPL129W |
| Hda1 | YNL021W | Mlp1 | YKR095W | Sap30 | YMR263W | Ubp8 | YMR223W |
| Hda2 | YDR295C | Mlp2 | YIL149C | Sas2 | YMR127C | Uip3 | YAR027W |
| Hda3 | YPR179C | Nap1 | YKR048C | Sas3 | YBL052C | Ume1 | YPL139C |
| Hfi1 | YPL254W | Nat1 | YDL040C | Sas4 | YDR181C | Ume6 | YDR207C |
| Hos2 | YGL194C | Nat3 | YPR131C | Sas5 | YOR213C | Vps71 | YML041C |
| Hos3 | YPL116W | Nat4 | YMR069W | Sdc1 | YDR469W | Vps72 | YDR485C |
| Hos4 | YIL112W | Nat5 | YOR253W | Sds3 | YIL084C | Vps75 | YNL246W |
| Hpa3 | YEL066W | Ngg1 | YDR176W | Set2 | YJL168C | Yaf9 | YNL107W |
| Hsl7 | YBR133C | Nhp10 | YDL002C | Set3 | YKR029C | Ydl089w | Ydl089w |
| Hst1 | YOL068C | Nto1 | YPR031W | Sgf11 | YPL047W | Yng1 | YOR064C |
| | | Nup100 | YKL068W | Sgf73 | YGL066W | Ypr174c | Ypr174c |
