# Supplementary material for: One-Cell Doubling Evaluation by Living Arrays of Yeast, ODELAY!
Source: G3 (Bethesda). 2016 Nov 16;7(1):279–88. doi: 10.1534/g3.116.037044 (PMC5217116; doi:10.1534/g3.116.037044)
Supplement: Supplementary file 5 [file 279TableS2.pdf]

Table S2: Comparison of ODELAY with other growth phenotype assays

Method	Assay type	Measure	Temporal analysis	Pixel resolution	Measures Lag	Measures Doubling Time	Measures Carrying Capacity	Measures Clonal Heterogeneity	Reported Throughput
BioScreen™	Liquid Culture	Optical Density at 600nm	Yes	NA	Estimated	Directly	Directly	No	200 growth curves /instrument
SGA –Boone Lab	Colony Pinning onto Agar	Patch Area	No	24 μm min ~42 μm typical	No	No	Directly	No	1536 patches /plate typical
ScanLag	Colony Pinning onto Agar	Patch Area	Yes	24 μm min ~42 μm typical	Estimated	Directly	Directly	No	1536 /plate typical
Ultra-High Density Omics	Colony Pinning onto Agar	Patch Area	Yes	~30 μm/pixel	No	Directly	Directly	No	6144 growth curves /plate
ScanoMatic	Colony Pinning to Agar	Patch Area	Yes	24 μm min ~42 μm typical	Estimated	Directly	Directly	No	1536 growth curves /plate typical
Bet Hedging	Liquid Culture	Multiple Colony Area	Yes	0.65μm Est	No	Directly	No	Directly	96 strains 1-1000 growth curves per strain
ODELAY	Single cell spotting onto Agar	Multiple Colony Area	Yes	0.65μm	Directly	Directly	Directly	Directly	96 strains 1-1000 growth curves per strain
